# Durability and Biological Response of a New Posterior Dynamic Stabilization System Using Polyethylene with Vitamin E

**DOI:** 10.1155/2018/5785708

**Published:** 2018-09-25

**Authors:** Koji Matsumoto, Yasuaki Tokuhashi

**Affiliations:** Department of Orthopaedic Surgery, Nihon University Itabashi Hospital, 30-1 Oyaguchikamimati Itabashi-ku, Tokyo 173-8610, Japan

## Abstract

**Objective:**

The purpose of this study was to evaluate the durability and biological response of a new Posterior Dynamic Stabilization system using polyethylene with vitamin E on the sliding surface.

**Summary of Background Data:**

The use of polyethylene with vitamin E on the sliding surface in Posterior Dynamic Stabilization has not been reported previously.

**Methods:**

A developed pedicle screw-based Posterior Dynamic system consists of four parts: a set screw, a rod, a ball, and a pedicle screw. The rod is inserted into the through hole of the ball, and the ball is sandwiched by the set screw.* (1) Fatigue Wear Test*. Testing was conducted under a dynamic compressive load of 50N at a speed of 1 Hz for 1 million cycles. We examined the loss of polyethylene due to abrasion in 3 units.* (2) Biological Response in Pigs*. In two pigs, a new pedicle screw and a conventional pedicle screw were inserted in L2 and L3/4, and L4 and L2/3, respectively. After breeding for 6 months, autopsies were performed. CT imaging was used to evaluate bone union of the facet joint. Abrasive specimens were prepared, and abrasion powder and inflammatory cell infiltration were evaluated microscopically.

**Results:**

The average loss of polyethylene due to abrasion was -0.01 mg. In all units, polyethylene showed a decrease of 0.1 mm or less at the contact point with the set screw. The facet joints between the conventional screws exhibited bone fusion, but the facet joint between the conventional and the new screw retained mobility with no bony fusion. No abrasion powder was found and inflammatory cell infiltration was only minimally observed.

**Conclusion:**

The new Posterior Dynamic Stabilization system exhibited a high level of durability and biological safety.

## 1. Introduction

At present, spinal fusion is widely performed. However, spinal fusion surgery increases the load and stress caused by movement in the adjacent segment. The occurrence of adjacent segment disease (ASD) after spinal fusion is reported as 4.8-92.2% [1] and is considered an inevitable complication after spinal fusion surgery. In order to avoid these complications, various Dynamic Stabilization systems have been developed [2–5]. Nonfusion stabilization has been reported as preventing ASD in two meta-analyses [6, 7]. However, these systems are problematic in terms of durability and biological reactions caused by abrasion powder [8–13]. Artificial joints for the knee and hip using polyethylene on the sliding surface have been developed and applied clinically and have achieved good results. We have developed a new pedicle screw system which features polyethylene on the sliding surface. The system uses vitamin E-containing polyethylene, which has been used on the sliding surface for knee and hip artificial joint replacement surgery and is said to be resistant to oxidative stress and abrasion [10, 12]. Until now, no system using polyethylene with vitamin E on the sliding surface in Posterior Dynamic Stabilization has been reported. The present study evaluates the durability and the biological response of this new system.

## 2. Materials and Methods

**Figure 1:**
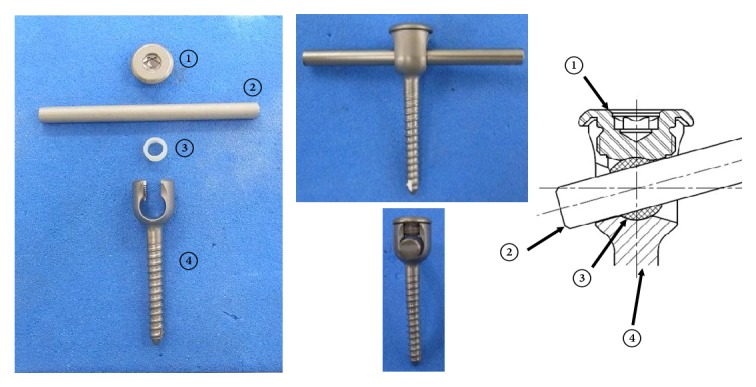
A new Posterior Dynamic Stabilization system using polyethylene with vitamin E. ① A set screw, ② a rod, ③ a ball of polyethylene, and ④ a pedicle screw. The rod is inserted into the through hole of the ball and the ball is sandwiched by the set screw.

New Posterior Dynamic Stabilization system: a major feature of this new pedicle screw system is that the relationship between the vertebral body and the rod is not completely fixed but semi-fixed. It consists of four parts: a set screw, a rod, a ball, and a pedicle screw. The pedicle screw and set screw are made of Ti - 6Al - 4V alloy, and the ball is made of cross-linked UHMWPE (ultra high molecular weight polyethylene) containing vitamin E (Teijin Nakashima Medical, Blend - E XL). It was achieved by irradiation with a 10MeV electron beam at 300kGy. The ball's diameter is 8 mm and a through hole of 5.5 mm matching the rod diameter exists in the center of the ball. The rod is inserted into the through hole of the ball and fitted to the U-shaped groove of the pedicle screw, and the ball is sandwiched by the set screw. An existing product (Stryker Xia *φ* 5.5 mm Ti - 6 Al - 4 V) is used for the rod. The pedicle screw has a range of movement of up to 15° on one side around the ball and also allows movement between the rod and the ball. The sliding surface is made of metal - polyethylene, and the two opposing surfaces on which the ball slides feature mirror surface processing. One is a pedicle screw U-shaped groove; another is inside the set screw. Each contact surface has a concave shape because contact is made with a spherical diameter of 8 mm (Figure 1).

### 2.1. Fatigue Wear Test

**Figure 2:**
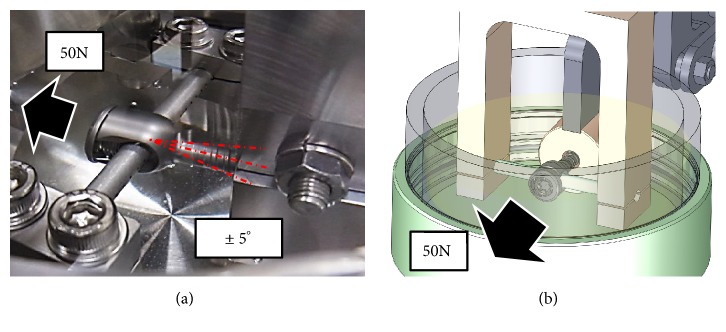
(a) Photo of the durability testing machine: testing was conducted under a dynamic compressive load of 50 N at a speed of 1 Hz for 1 million cycles. (b) Pattern diagram of the durability test machine.

The new pedicle screw system was fixed to the testing machine (Mini Bionix: MTS Japan) (Figure 2). Testing was conducted under a dynamic compressive load of 50N at a speed of 1Hz for 1 million cycles. We examined the loss of polyethylene due to abrasion and the presence or absence of system breakage of 3 units. The test room temperature was 20.3 to 23.1°C and the humidity was 25 to 35%. A calf serum solution with a total protein mass of 20 g/l was used as the lubricating liquid [14]. The weight of the ball was measured with an electronic balance (A&D). The loss of polyethylene was calculated as the weight difference between the polyethylene at the end of the test and another polyethylene sample immersed in lubrication during the same period as the test. The appearance of the ball, the sliding surface of the set screw, the sliding surface of the main body screw, and the appearance of the rod were observed with a digital microscope (Keyence), and the shape change of the ball was observed with a three-dimensional digitizer (SOLUTIONIX).

### 2.2. Biological Response in Pigs

**Figure 3:**
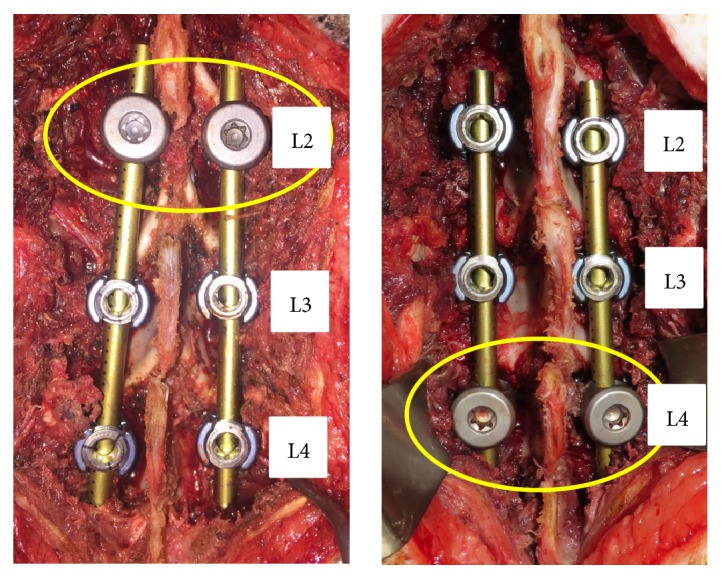
Pig intraoperative photo. A new pedicle screw and a conventional pedicle screw were inserted in L2 and L3,4, and L4 and L2,3, respectively.

The experiment was conducted on two mini pigs with an age and weight of 3 years, 7 months and 47.4 kg, and 2 years, 8 months and 48.3 kg, respectively. The two mini pigs were anesthetized and screws inserted the pedicles of both sides of L2.3.4. In the first pig, a new pedicle screw was inserted in L2 and a conventional pedicle screw (5.5 × 40 mm Stryker Xia) was inserted in L3,4. In the second pig, a new pedicle screw was inserted in L4 and a conventional pedicle screw was inserted in L2,3. Screws were fixed with a 5.5 mm rod (Stryker Xia) (Figure 3). After breeding for 6 months, autopsies were performed and vertebral bodies, screw, and rod were removed in one piece. CT imaging was used to evaluate bone union of the facet joint. In addition, abrasive specimens were prepared. After Villanueva bone staining and Toluidine blue staining, the presence or absence of polyethylene and metal abrasion powder and bone soft tissue surrounding the screw were evaluated histologically under a microscope. This study has been approved by the Ethics Committee of Nihon University School of Medicine.

## 3. Results

### 3.1. Fatigue Wear Test

**Figure 4:**
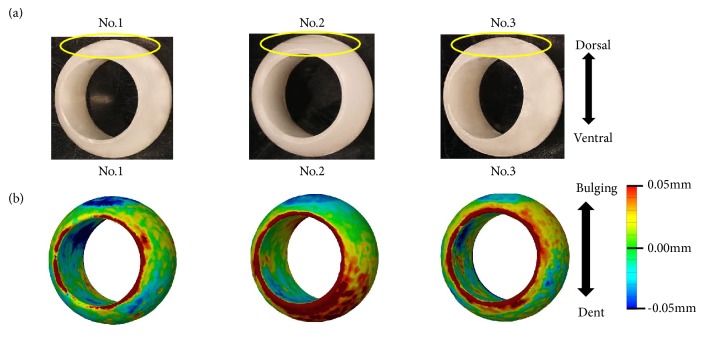
(a) Appearance of polyethylene ball: in all units, a depression was found at the contact points with the set screw. (b) Shape change of polyethylene ball: the depression seen in all units was less than 0.1 mm.

The loss of polyethylene was 0.02 mg for Unit No. 1, 0.01 mg for Unit No. 2, and -0.06 mg for Unit No. 3. The average loss of polyethylene due to abrasion was -0.01 mg (Table 1). All units showed a polyethylene decrease of 0.1 mm or less at the contact point with the set screw (Figure 4). No obvious damage was observed on the sliding surface of the pedicle screw, the rod, and the set screw.

### 3.2. Biological Response in the Facet Joint

**Figure 5:**
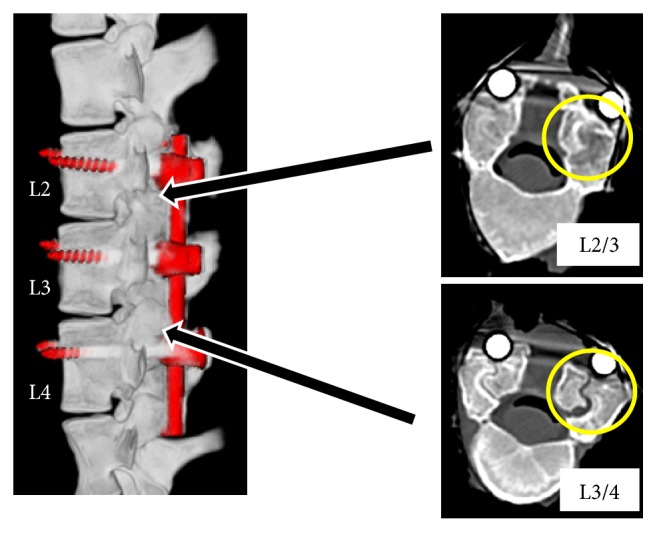
CT image of the spine removed from a pig. The joint space disappears as a result of rigid fixation and bone fusion (L2/3). The joint space is retained as a result of Dynamic Stabilization (L3/4).

The facet joints between the conventional screws exhibited bone fusion, but the facet joint between the conventional and the new screw retained mobility with no bony fusion (Figure 5).

### 3.3. Biological Response in Bone Soft Tissue Surrounding the Pedicle Screw

No polyethylene or metal abrasion powder was found in the tissue.

Abnormal osteolysis was not observed and inflammatory cell infiltration was only minimally observed in the bone and soft tissue surrounding the screw.

**Figure 6:**
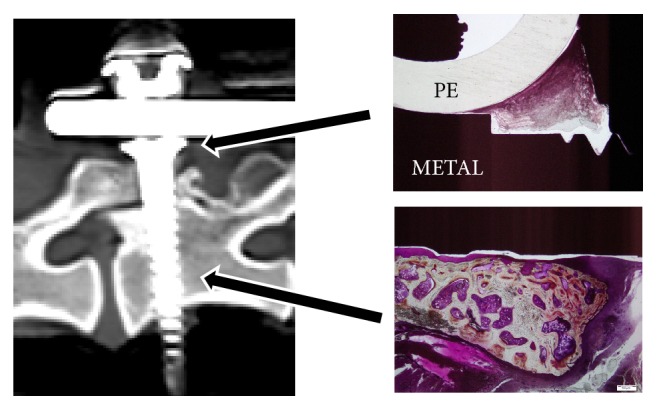
Microscopic image (Villanueva bone stain: photograph magnification 20 times). Polyethylene (PE) and metal abrasion powder were not found in the soft tissue around the screw head. Abnormal osteolysis was not observed in bone tissue around the screw, and inflammatory cell infiltration was only minimally observed.

Aseptic lymphocyte-dominant vasculitis-associated lesion (ALVAL), a reactivity change caused by abrasion powder, was not observed (Figure 6).

## 4. Discussion

In order to avoid the complication of adjacent segmental disease after spinal fusion surgery, various systems that preserve intervertebral disc mobility without rigid intervertebral fusion have been developed [2]. However, none of these have proved to be ideal. Existing systems have a sliding surface of metal on metal, but metal on metal generates metallic abrasive powder. Such systems have been reported to cause an adverse reaction due to metal debris (ARMD) mainly based on lymphocyte accumulation and tissue necrosis [15, 16]. Osteolysis, a result of the biological reaction caused by polyethylene abrasive powder, has also been reported in a system using a sliding surface of metal on polyethylene [17]. Therefore, it is important to use polyethylene which has excellent durability against abrasion on the sliding surface. Although artificial joints using polyethylene on sliding surfaces have been developed for various joints, Posterior Dynamic Stabilization using polyethylene on the sliding surface in the field of the spine has not been reported. We have developed a new Posterior Dynamic Stabilization system using vitamin E-containing high molecular weight polyethylene, which has been reported to have excellent durability in impingement and friction under oxidative stress [10, 12]. In the durability test of the present study, the decrease in polyethylene due to abrasion was very small, and deformation in the polyethylene was also mild. However, in Unit No. 3, polyethylene weight exhibited an increase after the test. This may have been caused by the absorption of moisture and oil texts in the lubricating liquid. We believe that polyethylene wear in Unit No. 3 was as minimal as it was in the other two units, which would explain the increase seen in Unit No. 3 as being the result of absorption. In addition, since the polyethylene sample used to calculate the loss of polyethylene was not loaded, the conditions for absorption were different from that of the unit undergoing the wear test. Although this was a limitation in our research, it seems to be a measurement error indicating a very small degree of wear. In addition, no damage was observed on the pedicle screw, the rod, or the set screw including the sliding surface. It was possible to reduce the occurrence of abrasion powder to a very low level while confirming the durability of the new system. Furthermore, in the animal experiment using two mini pigs, no polyethylene or metal wear powder was found in the tissue. Inflammatory cell infiltration was only minimally observed and osteolysis and ARMD were not observed. These results indicate the safety of the new pedicle screw system in vivo. The facet joints between the conventional screws exhibited bone fusion, but the facet joint between the conventional and the new screw retained mobility with no bony fusion. These results indicate that new Posterior Dynamic Stabilization system functions in vivo and supports the possibility of its clinical application. Artificial joints using polyethylene for sliding surfaces in the knee, elbow, and hip joints are widely used, and the ability to apply such mechanisms to the sliding surface of the spine is a subject that will continue to see further development in the future. The most important findings of this study are that polyethylene was used for the first time on the sliding surface in Posterior Dynamic Stabilization and that a high degree of durability and biological safety were attained. However, it is recognized that this system is the first prototype and that there is still much room for research and development. The limitation of this study is the difference in the vertebral body characteristics in mini pigs and humans. This system is limited in the braking force of flexion-extension although there is a braking force in the anterior-posterior direction, and the long-term performance is unknown. Further development of instruments and long-term observation are desired.

## 5. Conclusion

A new Posterior Dynamic Stabilization system using polyethylene on the sliding surface exhibited a high level of durability and biological safety.

## Figures and Tables

**Table 1:** Abrasion amount of polyethylene.

No. 1	No. 2	No. 3	Average
0.02 mg	0.01 mg	-0.06 mg	-0.01 mg

## Data Availability

The data used to support the findings of this study are available from the corresponding author upon request.
